# Normalizing acronyms and abbreviations to aid patient understanding of clinical texts: ShARe/CLEF eHealth Challenge 2013, Task 2

**DOI:** 10.1186/s13326-016-0084-y

**Published:** 2016-07-01

**Authors:** Danielle L. Mowery, Brett R. South, Lee Christensen, Jianwei Leng, Laura-Maria Peltonen, Sanna Salanterä, Hanna Suominen, David Martinez, Sumithra Velupillai, Noémie Elhadad, Guergana Savova, Sameer Pradhan, Wendy W. Chapman

**Affiliations:** Department of Biomedical Informatics, University of Utah, Salt Lake City, UT USA; Nursing Science, University of Turku, and Turku University Hospital, Turku, Finland; Data61, CSIRO, The Australian National University, University of Canberra, and University of Turku, Locked Bag 8001, Canberra, 2601 ACT Australia; MedWhat.com, San Francisco, CA USA; University of Melbourne, Parkville, VIC Australia; Department of Computer and Systems Sciences (DSV), Stockholm University, Stockholm, Sweden; Department of Biomedical Informatics, Columbia University, New York, NY USA; Boston Children’s Hospital, Harvard Medical School, Boston, MA USA

**Keywords:** Natural language processing, Acronyms, Abbreviations, Consumer health information, Unified Medical Language System

## Abstract

**Background:**

The ShARe/CLEF eHealth challenge lab aims to stimulate development of natural language processing and information retrieval technologies to aid patients in understanding their clinical reports. In clinical text, acronyms and abbreviations, also referenced as *short forms,* can be difficult for patients to understand. For one of three shared tasks in 2013 (Task 2), we generated a reference standard of clinical short forms normalized to the Unified Medical Language System. This reference standard can be used to improve patient understanding by linking to web sources with lay descriptions of annotated short forms or by substituting short forms with a more simplified, lay term.

**Methods:**

In this study, we evaluate 1) accuracy of participating systems’ normalizing short forms compared to a majority sense baseline approach, 2) performance of participants’ systems for short forms with variable majority sense distributions, and 3) report the accuracy of participating systems’ normalizing shared normalized concepts between the test set and the Consumer Health Vocabulary, a vocabulary of lay medical terms.

**Results:**

The best systems submitted by the five participating teams performed with accuracies ranging from 43 to 72 %. A majority sense baseline approach achieved the second best performance. The performance of participating systems for normalizing short forms with two or more senses with low ambiguity (majority sense greater than 80 %) ranged from 52 to 78 % accuracy, with two or more senses with moderate ambiguity (majority sense between 50 and 80 %) ranged from 23 to 57 % accuracy, and with two or more senses with high ambiguity (majority sense less than 50 %) ranged from 2 to 45 % accuracy. With respect to the ShARe test set, 69 % of short form annotations contained common concept unique identifiers with the Consumer Health Vocabulary. For these 2594 possible annotations, the performance of participating systems ranged from 50 to 75 % accuracy.

**Conclusion:**

Short form normalization continues to be a challenging problem. Short form normalization systems perform with moderate to reasonable accuracies. The Consumer Health Vocabulary could enrich its knowledge base with missed concept unique identifiers from the ShARe test set to further support patient understanding of unfamiliar medical terms.

## Background

International healthcare policies aim to improve patients’ access to their clinical record and involvement in their healthcare delivery, e.g. in the United States [1], in Australia [2], and in Finland [2]. These policies have motivated healthcare organizations to adopt patient-centered approaches e.g., the United States Open Notes project [3], some resulting in modest benefits and minimal risks [4–8].

Patient access to easy-to-understand, simple text in clinical reports is also stipulated in several countries by law. For instance, regulations in the United States [9] and European Union [10] state that patients should have access to their clinical information upon request [11]. United Kingdom guidelines describe best practices for patient access [12]. Laws and statutes in Sweden [13] and Finland [14] state that clinical notes must be explicit and comprehensive, including only well known, accepted concepts and abbreviations.

Automated tools for text simplification can help clinicians comply with regulations and improve information readability for patients. For instance, statistical approaches can identify, reduce, and disambiguate unfamiliar concepts. Specifically, unsupervised methods and statistical associations can automatically learn unfamiliar terms, identify potential semantic equivalents, and present lay terms or definitions [15–17]. Text simplification architectures can analyze, transform, and regenerate sentences for patients e.g., simplifying Wall Street Journal sentences for Aphasia patients [18]. In the biomedical domain, one text simplification tool reduces the semantic complexity of sentences conveying health content in biomedical articles by substituting unfamiliar medical concepts with synonyms or related terms, and the syntactic complexity by dividing longer sentences into shorter constructions [19]. In the clinical domain, a prototype translator reduces the semantic complexity of clinical texts by replacing abbreviations and other terms with consumer-friendly terms from the Consumer Health Vocabulary and explanatory phrases [20].

Making annotated corpora available to the natural language processing community through shared tasks can further stimulate development of technologies in this research area [21]. Like the Message Understanding Conference (MUC) [22], Text REtrieval Conference (TREC) [23, 24], Genome Information Acquisition (GENIA) [25, 26], and Informatics for Integrating Biology and the Bedside (i2b2) challenges [27–31], the 2013 Shared Annotated Resources/Conference and Labs of the Evaluation Forum (ShARe/CLEF) eHealth Challenge evaluated participant natural language processing systems against a manually-generated reference standard [32]. The 2013 ShARe/CLEF eHealth Challenge took initial steps toward facilitating patient understanding of clinical reports by identifying and normalizing mentions of diseases and disorders to a standardized vocabulary (Task 1) [33], by normalizing acronyms and abbreviations (Task 2) [34], and by retrieving documents from health and medicine websites for addressing patient-centric questions about diseases and disorders documented in clinical notes (Task 3) [35]. This paper describes studies related to Task 2.

We review acronym and abbreviation recognition in the context of text normalization. We are motivated by the need for creating an annotated corpus of acronyms and abbreviations to encourage the development of natural language processing tools that improve patient understanding and readability of clinical texts.

### Text processing for acronyms and abbreviations

Conceptually disambiguating the meaning of a word or phrase from clinical text often involves mapping to a standardized vocabulary [36]. For example, natural language processing tools that normalize words and phrases to Unified Medical Language System (UMLS) concept unique identifiers (CUIs) include IndexFinder [37], KnowledgeMap [38], MetaMap [39], Medical Language Extraction and Encoding System (MedLEE) [40] and clinical Text Analysis and Knowledge Extraction System (cTAKES) [41]. Acronyms and abbreviations are shortened words used to represent one or more concepts [42]. Acronyms are formed from the first letters of words in a meaningful phrase (‘BP’ = *Blood Pressure*) and can be pronounced as words (‘CABG’ = Coronary Artery Bypass Graft, pronounced *cabbage*) or letter-by-letter (‘TIA’ = Transient Ischemic Attack, pronounced *T-I-A*). Abbreviations are shortened derivations of a word or phrase (‘myocard infarc’ = *myocardial infarction*) and are generally pronounced as their expanded forms (‘myocard infarc’, pronounced *myocardial infarction*). We will refer to acronyms and abbreviations throughout the manuscript as short forms for brevity and to convey a mixture of both acronyms and abbreviations, including their lexical variants from the clinical text.

Accurate short form detection methods may handle various linguistic characteristics and phenomena associated with short form usage in text. Short forms are documented using different orthographic constructions including varied letter case (‘CAD’ vs ‘cad’). Punctuation can be applied to acronyms to represent one concept (‘b.i.d.’ means *twice a day*) or list many related concepts (‘m/r/g’ represents three heart sound concepts - *murmurs, rubs, gallops*). Syntactically, short forms may be conveyed using both singular and plural forms (‘TIA’/‘TIAs’) as well as possessives (‘Pt’s’). Syntactically, a short form can conceptually represent different long forms of the same concept and semantically, short forms may be polysemous, having different, but related word senses (‘LV’ can stand for an adjectival phrase like *left ventricular* or a noun phrase *left ventricle*). Short forms may be homonymous, having different, unrelated word senses within and across report genres (‘LV’ can stand for both *left ventricle* and *low volume* in an echocardiogram report, but would more likely stand for *lumbar vertebrae* in a radiology report). In fact, short form ambiguity can lead to unintended medical errors; therefore, many short form are banned from clinical document usage by the Joint Commission, in “*Do not use List: Abbreviations, Acronyms, and Symbols*” [43]. Short forms can occur with misspellings (‘myocrad infrac’ should be spelled ‘myocard infarc’) and can be concatenated with other words (‘70 year oldM’ = ‘70 year old M’). We developed an annotation schema and guidelines for human annotators that addressed the annotation of such examples from clinical text (described under Methods Annotation Schema).

### Text normalization

In general, text normalization, which may include short form (mention-level) boundary detection, word sense disambiguation, and named entity and event recognition, can be an important processing step for some clinical information extraction approaches. For example, in order to extract a disease and disorder mention and link it to information to help a patient’s understanding of the unfamiliar medical concept e.g., such as Abdominal tenderness from ‘ABD: Tender,’ a natural language processing system would need to 1) detect ‘ABD’ as a short form, 2) disambiguate ‘ABD’ as *Abdomen* not *Aged, Blind and Disabled,* 3) normalize ‘ABD’ to a concept in a controlled vocabulary (e.g., C0562238: Examination of the Abdomen)*,* 4) post-coordinate ‘ABD’ with the adjacent finding *tenderness* (e.g., C0234233: Tenderness) to define an event (e.g. C0232498: Abdominal tenderness), and finally 5) link it to a web-based information resource like Medline Plus. For the purposes of our assessment, we have focused on short form disambiguation (2) and normalization (3).

### Acronym and abbreviation detection and normalization

Early and ongoing work on aspects of short form detection and normalization focused on developing resources in the biomedical literature domain, in particular, MEDLINE abstracts. A common and reasonable, baseline approach to detecting and normalizing biomedical short forms is exploiting short form – long form patterns [44–46]. This method is advantageous because most short forms demonstrate no or low ambiguity and can be mapped to the most frequent sense usage. Furthermore, few short forms demonstrate moderate to high ambiguity due to polysemous and/or homonymous usage.

Researchers have also developed more sophisticated, high performing biomedical short form disambiguation modules by training supervised models and evaluating against MEDLINE corpora, e.g., Medstract Gold Standard Evaluation corpus (support vector machines: 98 % F1-measure [47], logistic regression: 81 % F1-measure [48]) and semi-supervised (AbbRE): 91 % F1-measure [49]). Further resources – databases and tools – for disambiguating biomedical short forms include Acronym Resolving General Heuristic (ARGH), Stanford Biomedical Abbreviation Database, AcroMed, and Simple and Robust Abbreviation Dictionary (SaRAD) [48, 50]. However, few resources exist for short form recognition from clinical texts.

Indeed, a comparison study of state-of-the-art clinical text normalization tools suggests that clinical short forms detection and normalization is still in its early stages [51]. This study determines that clinical short forms normalization tools generally demonstrate low to moderate performance - clinical Text Analysis and Knowledge Extraction System (F1-measure: 21 %), MetaMap (F1-measure: 35 %), and Medical Language Extraction and Encoding System (F1-measure: 71 %) [51]. Natural language processing systems can perform with low normalization scores due to multiple senses for a short form. The study suggests that the reason that the Medical Language Extraction and Encoding System outperforms MetaMap and clinical Text Analysis and Knowledge Extraction System for disambiguating ambiguous short form is due to its highly integrated clinical sense inventories [51]. Natural language processing researchers have successfully produced sense inventories and automated disambiguation modules using rule-based and machine learning-based approaches [52, 53]. For instance, a short form sense inventory was generated using regular expressions and morphological heuristics from 352,267 clinical notes and the most frequent short forms were manually mapped to three vocabularies – Stedman’s Medical Abbreviations, Acronyms & Symbols, the Unified Medical Language System, and Another Database of Abbreviations in Medline (ADAM) [52]. Such sense inventories were developed using features generated from the Internet, Medline, and Mayo clinical notes to train decision tree and maximum entropy classifiers for eight short forms [53]. Both decision tree (94 %) and maximum entropy (96 %) classifiers demonstrated more accurate short form classification than a majority sense baseline (71 %). Disambiguation modules focus on ambiguous word-senses of clinical short forms [54]. One disambiguation module uses a support vector machine trained with 125 samples that achieved high accuracy (over 90 %) for the 50 most frequent short forms with varied senses from a dataset of 604,944 clinical notes. In addition to support vector machines, decision trees and naïve bayes classifiers are able to disambiguate short forms with high accuracy (exceeding 90 %) using part-of-speech, unigram, and bigram features [55]. Semi-supervised (Specialist Lexicon Release of Abbreviations and Acronyms (LRABR) with multi-class support vector machine) and unsupervised (hierarchical clustering) approaches have also demonstrated moderate to excellent disambiguation performance [56]. Although rule-based and machine learning-based approaches can disambiguate short forms with multiple senses from a subset of data, more work can be done addressing a larger subset of short forms and report types. To enable further progress in this area, we have developed a corpus annotated with clinical short forms linked to normalized values.

With recent patient-centered initiatives, the focus of the 2013 ShARe/CLEF eHealth Challenge was to facilitate development of natural language processing applications that could be used to help patients understand the content of a clinical report, and Task 2 focused on normalization of short forms. We describe the performance of the participating systems at automatically normalizing clinical short forms to the Unified Medical Language System compared to a majority sense baseline, evaluate the performance of participating systems according to short form terms with variable majority sense distributions, and assess each participating systems’ performance for concepts shared between the ShARe test corpus and a vocabulary containing simplified health terms. The study extends the overview of all three tasks [32] and organizers’ working notes on Task 2 [34, 57] by focusing on Task 2 in significantly greater depth, focusing on 1) the difficulty of handling multiple short form senses, and 2) the utility of each participating system with respect to a vocabulary containing simplified health terms for potentially supporting patient understanding of clinical text.

## Methods

In this section, we describe the short form schema, dataset, shared task, sense categorization, and short form coverage using the Consumer Health Vocabulary.

### Annotation schema

We developed our annotation schema and guidelines using a top-down and bottom-up methodology. We applied top-down knowledge of text normalization by starting with an annotation approach focusing on clinical short forms described in [51, 58]. We added rules based on guidelines from Task 1 developed for disease and disorder annotation and refined these rules through feedback provided by a panel of four natural language processing experts (WWC, SP, NE, and GS) to develop an initial schema and guidelines. Annotation by two biomedical informatics students (DLM and BRS) on ten reports provided a bottom-up approach to validate these rules and clarify instructions through examples in the guidelines. For example, we applied a top-down rule derived from the Task 1 guidelines to exclude modifying information like *negation*, *history*, and *change* in the concept description (e.g., ‘*no* eff’). After annotating ten reports, we refined this rule with a bottom-up approach to include *anatomic locations, sidedness*, and *structures* within the short form span boundaries (e.g., ‘*bilat pleur* eff’) based on the data. We included *sections, diseases and disorders, signs and symptoms, diagnoses, procedures, devices, gender, healthcare unit names.* We excluded *medications, lab results, measurement units, non-medical short forms, severities, and salutations.* Annotators were also provided Task 1 disease and disorder annotations to help annotate short forms and interpret the annotation rules. For instance, annotators were provided the Task 1 annotation C0232498: Abdominal tenderness for the finding “ABD: Tender.” and encouraged to use this knowledge to assign “ABD” as *Abdomen* rather than *Aged, Blind and Disabled.* Similar to Task 1, annotators were instructed to assign the label ‘CUI-less’ to a short form span when no appropriate concept description existed in the vocabulary. For Task 2, annotators mapped short form spans to the Unified Medical Language System. The final schema contained inclusion and exclusion rules for 1) identifying the character spans of short form terms in the corpus (boundary detection) and 2) normalizing short forms to CUIs from the Unified Medical Language System 2012 using an application program interface call within an annotation tool (extensible Human Oracle Suite of Tools - eHOST). The final guidelines can be viewed in detail on the ShARe website [59].

### Dataset

For this IRB-approved study, we leveraged the ShARe corpus, a subset of de-identified discharge summary, electrocardiogram, echocardiogram, and radiology reports from about 30,000 ICU patients provided by the Multiparameter Intelligent Monitoring in Intensive Care (MIMIC) II database [60]. As part of ShARe/CLEF eHealth Challenge Task 1 [59], 298 clinical reports were split into training (*n* = 199 reports) and test (*n* = 99 reports) sets and annotated for disease and disorder mentions and their Systematized Nomenclature Of MEDicine Clinical Terms (SNOMED CT) codes by two professional medical coders. We maintained these splits and provided the Task 1 corpus to Task 2 annotators to annotate clinical short forms along with their normalized values. We achieved high inter-annotator agreement of 91 % for the test dataset between annotations that were reviewed and adjudicated by a biomedical informaticist and a respiratory therapist. We further characterize the corpus development and inter-annotator agreement in [32–34].

### ShARe/CLEF eHealth challenge shared task 2 - participating teams

The annotated ShARe corpus was released as part of the 2013 ShARe/CLEF eHealth Evaluation Challenge [61]. Two training sets were provided containing short form spans and CUIs. Participants were instructed to develop a natural language processing normalization system to predict the CUI for each provided short form span in the test dataset. In summary, five teams – *UTHealthCCB* [62]*, LIMSI* [63]*, TeamHealthLanguageLABS* [64], *THCIB* [65]*,* and *WVU* [66] - submitted systems for Task 2. Four teams approached this task using machine learning-based methods: three teams built conditional random field classifiers [63, 64, 66] and one team used support vector machines [62]. The teams used a variety of features including lexical, morphological, and structural features from the Unified Medical Language System, Systematized Nomenclature Of MEDicine Clinical Terms, clinical Text Analysis and Knowledge Extraction System, and gazetteers. One team built a rule-based system combining clinical Text Analysis and Knowledge Extraction System and rules developed from the training data [65]. Specifically, the five participating teams developed the following short form normalization solutions:

- ➢ *UTHealthCCB* [62] applied one of four different sense tagging methods based on short form characteristics of frequency (high or low) and ambiguity (present or not): 1) a trained support vector machine mapped high frequency and ambiguous short forms, 2) a majority sense method mapped high frequency and unambiguous short forms, 3) a vector space model mapped all low frequency short forms, and 4) a Unified Medical Language System Terminology Services Application Programming Interface mapped any unseen short forms.
- ➢ *LIMSI* [63] applied clinical Text Analysis and Knowledge Extraction System and MetaMap to extract features - lexical and morphological (unigrams, short form terms, and token characteristics), syntactic (unigrams and bigrams part of speech), document (report and section types), semantic (MetaMap semantic type and CUI), and Wikipedia (semantic category) features. Many features included a context window of 1-3 tokens. These features were used to train a linear-chain conditional random field classifier using Wapiti.
- ➢ *TeamHealthLanguageLABS* [64] trained a linear-chain conditional random field classifier using context (bigram window), lexical (Lexicon Management System terms), grammatical (lemma, part of speech and chunk), ring-fence (complex and compound short forms) and Systematized Nomenclature Of MEDicine Clinical Terms (terms, concept id and category) features to identify short forms. A sequence of gazetteers applied the optimal CUI mapping based on possible expansions, usage frequency, and token contexts.
- ➢ *THCIB* [65] developed a rule-based system combining clinical Text Analysis and Knowledge Extraction System with custom short form and full name dictionaries developed from the training set as well as the STANDS4 online medical dictionary.
- ➢ *WVU* [66] trained a linear-chain conditional random field algorithm from the Factorie toolkit using a dictionary of short forms generated from the training data, Unified Medical Language System data sets, and general websites.

### System evaluation metrics

We compared each participating system predictions against the short form annotations in the test set using *accuracy* defined as the count of correct short forms divided by the total count of the reference standard short form annotations [67]. A system short form was correct if the assigned CUI matched the reference standard CUI*.* Participating teams were allowed to submit two systems each.

### Majority sense baseline

From the training data, we developed a majority sense baseline classifier, as this approach has been successful in other biomedical short form studies [44–46]. Based on the training data annotations, for which each short form annotation contains the short form span offset, term, and Systematized Nomenclature Of MEDicine Clinical Terms CUI, we generated a majority sense dictionary using frequency counts for each CUI associated with a unique short form term (converted to lower-case). The dictionary was structured as a list sorted first by CUI frequency and the most frequent CUI value was selected. For example, the short form term “ca” contains 2 unique CUI labels representing C0006826: Malignant Neoplasms: 5 or C0443758: Carbohydrate antigen: 1. If we observed “ca” as the short form term in the test set, we selected the most frequent CUI value for “ca” C0006826 as the normalization value (based on the frequency of the training set annotations); otherwise the short form term was assigned ‘CUI-less’. If the CUI were equally probable, we randomly selected the CUI to be used in the sense dictionary. For example, for the short form term “lle” we randomly selected C0239340 from the following CUI list: [C0230416: Left lower extremity: 1, C0239340: Edema of lower limbs: 1]. We compared the majority sense baseline and participant system accuracy scores for statistical significant differences using random shuffling [68].

### Sense prediction evaluation

We report the proportion of annotations from the test set for which a short form term has one unique sense versus two or more senses (CUI normalization values or CUI-less). Applying a discretization method [55], we report the majority sense distributions annotated for each of the top ten most frequent short form terms containing two or more senses and variable distributions across value sets. Furthermore, we assessed each participating team’s system performance according to the sense distribution categories below, which were defined to characterize the ambiguity of short form terms in the test dataset [55]:

- ➢ no ambiguity: short form terms with 1 unique sense
- ➢ low ambiguity: short form terms with > = 2 senses, majority sense >80 %
- ➢ moderate ambiguity: short form terms with > =2 senses, majority sense 50–80 %
- ➢ high ambiguity: short form terms with > = 2 senses, majority sense <50 %

### Consumer health vocabulary coverage

We evaluated the coverage of short form concepts and annotations from the ShARe corpus against a vocabulary of simplified, consumer-friendly terms, the Consumer Health Vocabulary [69] developed by Zeng and colleagues [66]. The Consumer Health Vocabulary provides lay terms for clinical concepts and contains a mapping to Unified Medical Language System preferred terms for each Consumer Health Vocabulary term. We queried each Unified Medical Language System CUI against the Consumer Health Vocabulary concept terms flat file from the Consumer Health Vocabulary website [70] to determine how frequently the preferred term was the same both in the Unified Medical Language System and the Consumer Health Vocabulary, and how frequently they differed.

For the test set, we report the prevalence of unique short form CUIs in the ShARe corpus and Consumer Health Vocabulary. We report the proportion of the Consumer Health Vocabulary concepts that provide a different preferred name than the preferred name in the Unified Medical Language System as mapped in the Consumer Health Vocabulary resource. For example, the patient-friendly term CT scan may be preferred over the clinical-friendly preferred term X-Ray Computed Tomography. From the test set, we also evaluated the coverage of short forms found in each vocabulary using *recall*, with true positives (TP) defined as a ShARe short form occurring in the vocabulary and false negatives (FN) defined as a ShARe short form not occurring in the vocabulary. Of annotations represented by CUIs shared by both the test set and the Consumer Health Vocabulary, we evaluated how well each participants’ system completed the normalization task using *accuracy*, with a TP defined as an short form correctly normalized to a shared ShARe/Consumer Health Vocabulary CUI and a FN defined as an short form missed or incorrectly normalized to a shared ShARe/Consumer Health Vocabulary CUI.

## Results

We characterized the ShARe short form corpus, assessed participants’ systems, reported majority sense distributions for the most prevalent terms, assessed participants’ systems for each majority sense distribution category, evaluated the coverage of short form concepts against the Consumer Health Vocabulary, and evaluated how well each participants’ system could normalize short forms with shared CUIs between the test set and the Consumer Health Vocabulary.

### Test corpus

On the test set of 99 clinical texts, we observed 3774 short form annotations, 603 unique terms, and 707 unique normalization values (CUIs and CUI-less). Six percent (221/3774) of short form annotations were assigned CUI-less.

### ShARe/CLEF eHealth challenge shared task 2 - system performances

Results for the participating systems and the majority sense baseline for normalizing short forms in the test set are summarized in Table 1. Although there were only 3774 observations in the test set, a total of 4892 unique annotations were submitted among participating teams. As a result of creating end-to-end systems (i.e. also predicting short form spans), several teams were missing annotations – from 163 (LIMSI.1) to 1415 (TeamWVU.1). UTHealthCCB had the highest accuracy (71.9). We compared the performance of the majority sense baseline against the performance of the top-performing system, UTHealthCCB.B.1. The majority sense baseline achieved an accuracy of 69.6. about 3 percentage points lower than the UTHealthCCB.B.1 system. However, the majority sense baseline outperformed the second ranked system from the same team, UTHealthCCB.B.2.

**Table 1:** ^a^Participant system performances from [32, 34] compared against a majority sense baseline performance

Short form normalization system	Unique predictions by the system	Annotations comparable with reference standard	Accuracy
^a^UTHealthCCB.B.1	3,774	3,774	71.9*
Majority Sense Baseline	3,774	3,774	69.6
^a^UTHealthCCB.B.2	3,774	3,774	68.3
^a^LIMSI.1	3,896	3,611	66.4
^a^THCIB.B.1	3,774	3,774	65.7*
^a^TeamHealthLanguageLABS	2,987	2,633	46.7*
^a^WVU.1	3,068	2,359	42.6

*Indicates that the difference in accuracy is statistically significant with the system immediately below (*p* < 0.01)

### Sense prediction evaluation

We observed that 603 unique terms from a total of 2095 (55 %) short form annotations in the test data have no ambiguity (1 unique sense); 135 unique terms from 1679 (45 %) annotations have two or more normalization values (CUI or CUI-less). Of the short forms with two or more normalization values, 47 unique terms, from 971 (26 %) annotations, have low ambiguity (equal or greater than 80 % majority sense); 80 unique terms, from 641 (17 %) annotations, have moderate ambiguity (50 to 80 % majority sense); and 8 unique terms from 67 (2 %) annotations, have high ambiguity (less than 50 % majority sense). In Table 2, we enumerate the top ten most frequent short form terms and their majority sense distributions for cases when two or more senses are observed according to ambiguity classes.

**Table 2:** Top 10 most frequent lexical variants with two or more senses according to distribution type

Short form term	Total count	Senses according to concept unique identifiers	Distribution of senses
*Low ambiguity*			
‘pt’	137	C0030705: Patients	89 %
		C0949766: Physical therapy procedure	4 %
		C0086835: Structure of the posterior tibial artery	4 %
		3 more senses	8 %
‘ct’	82	C0040405: X-Ray computed tomography	95 %
		C1274037: Cardiothoracic surgery	2 %
		C0008034: Thoracic drain	2 %
		1 more sense	1 %
‘m’	62	C0024554: Male gender	81 %
		C0018808: Heart murmur	16 %
		C0026591: Mother	2 %
		1 more sense	2 %
‘ekg’	41	C0013798: Electrocardiogram	98 %
		C1623258: Electrocardiographic procedure	2 %
‘f’	37	C0015780: Female	92 %
		C0015967: Fever	5 %
		CUI-less	3 %
‘cath’	33	C0007430: Catheterization	97 %
		C0085590: Catheter	3 %
‘lad’	33	C0226032: Anterior descending branch of left coronary artery	85 %
		C0497156: Lymphadenopathy	15 %
‘pcp’	31	C0033131: Primary care physicians	84 %
		C0032305: Pneumonia, Pneumocystis carinii	16 %
‘cad’	31	C1956346: Coronary artery disease	97 %
		C0010068: Coronary heart disease	3 %
‘abd’	29	C0562238: Examination of abdomen	90 %
		C0000726: Abdominal	10 %
*Moderate ambiguity*			
‘bp’	53	C1271104: Blood pressure finding	68 %
		C0005823: Blood pressure	32 %
‘r’	43	C0205090: Right	58 %
		C0232267: Pericardial rub	23 %
		C0035508: Rhonchi	11 %
		2 more senses	9 %
‘hr’	40	C0577802: Finding of heart rate	68 %
		C0018810: Heart rate	33 %
‘neuro’	34	C0027853: Neurologic examination	79 %
		C0205494: Neurologic (qualifier value)	6 %
		C0221571: Nervous system problem	6 %
		3 more senses	9 %
‘pod’	28	CUI-less	79 %
		C0032790: Postoperative period	21 %
‘ra’	26	C2709070: On room air	62 %
		C0225844: Right sided atrium	35 %
		C0456165: Right atrial pressure	4 %
‘bs’	26	C0232693: Bowel sounds	77 %
		C0035234: Respiratory Sounds	23 %
‘pa’	19	C1996865: Postero-anterior	53 %
		C0034052: Pulmonary artery structure	37 %
		C0428642: Pulmonary artery pressure	11 %
‘rrr’	18	C0232185: Cardiac rhythm AND/OR rate finding	67 %
		C0232188: Normal heart right	28 %
		C0513693: Monitor rate, rhythm, depth, and effort of respirations	6 %
‘mr’	18	C0026266: Mitral valve insufficiency	78 %
		C0024485: Magnetic resonance imaging	22 %
*High ambiguity*			
‘c’	25	C0010520: Cyanosis of skin	32 %
		C0149651: Clubbing	32 %
		C0205064: Cervical	24 %
		2 more senses	12 %
‘trach’	9	C0040590: Tracheostomy procedure	33 %
		C0184159: Tracheostomy tube	33 %
		C0040591: Tracheotomy procedure	11 %
		2 more senses	22 %
‘meds’	9	C0013227: Pharmaceutical preparations	44 %
		C0025118: Medicine	33 %
		C0033081: Drug prescriptions	22 %
‘cont’	8	C0549178: Continuous	38 %
		CUI-less	38 %
		C0584669: Recommendation to continue with treatment	13 %
		1 more sense	13 %
‘v’	6	C0042963: Vomiting	33 %
		C0348013: Venous	33 %
		C2228490: Examination of trigeminal nerve	33 %
‘d/c’	3	C0030685: Patient discharge	33 %
		C1444662: Discontinued	33 %
		C1548175: On discharge	33 %
‘pos’	3	C0205531: Oral route	33 %
		C0518037: Oral food intake	33 %
		C1446409: Positive	33 %
‘cvp’	3	C0199666: Measurement of central venous pressure	33 %
		C0428640: Central venous pressure	33 %
		C1321771: Central venous pressure finding	33 %

**Fig. 1:**
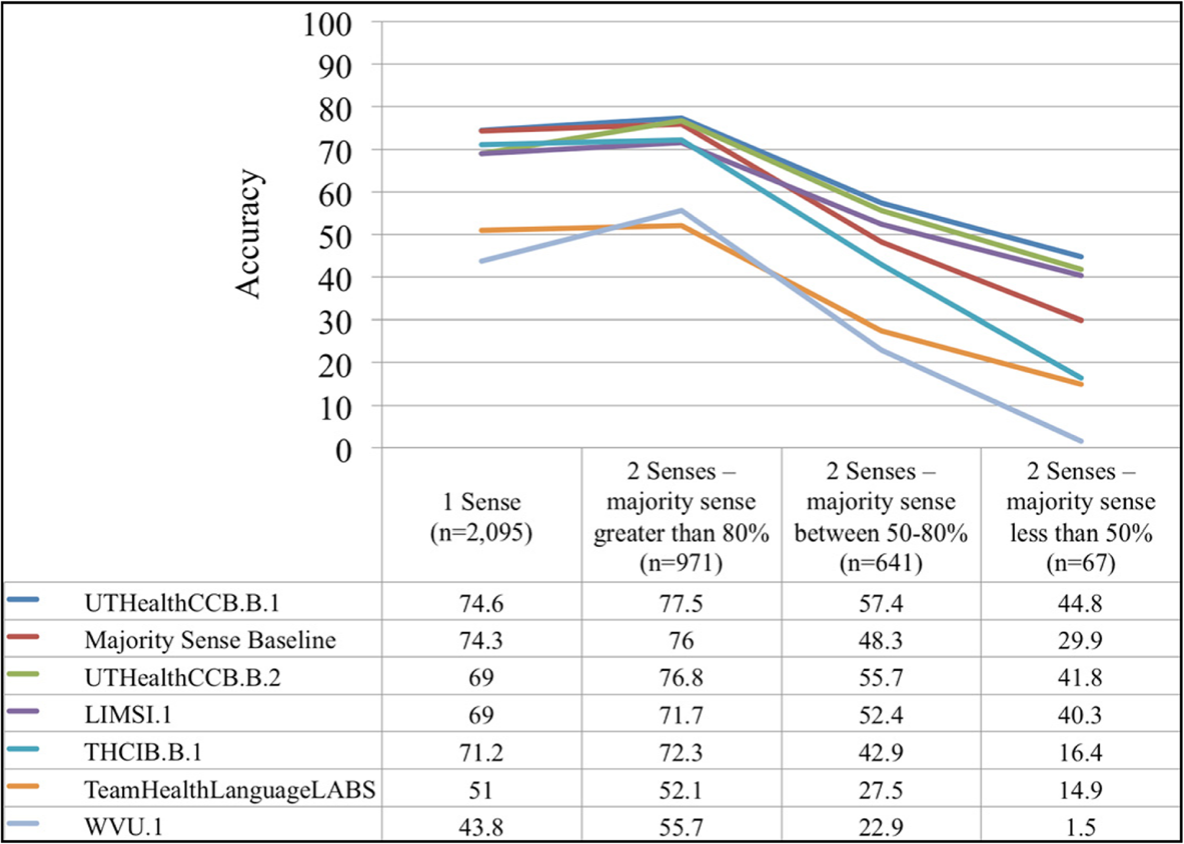
Accuracies of participating systems and Majority Sense Baseline for each majority sense distribution category

In terms of overall system performance, UTHealthCCB.B.1 achieved the highest accuracy across all sense categories (Fig. 1). The performance of participating systems for normalizing short form terms with no ambiguity compared to low ambiguity short form terms ranged from a slight increase in accuracy of 1.17 to 1.19 points. The performance of participating systems for normalizing low ambiguity short form terms compared to high ambiguity short form terms ranged from a decrease in accuracy of 31.4 to 55.9 points.

### Consumer health vocabulary coverage

The Consumer Health Vocabulary consists of 158,519 terms and 57,819 unique CUIs. The ShARe/CLEF short form test set consists of 860 unique terms and 706 unique Unified Medical Language System CUIs. We observed 66 % (466/707) of unique CUIs from the ShARe test set in the Consumer Health Vocabulary. Of the shared CUIs, 54 % (250/466) had a Consumer Health Vocabulary preferred term. For instance, C0027051: Myocardial Infarction occurs with a Consumer Health Vocabulary preferred name heart attack. We determined that 52 % (129/250) of the shared CUIs have a Consumer Health Vocabulary preferred term (patient-friendly name) that differed from the Unified Medical Language System preferred term (clinically-friendly name). For instance, C0013516: Echocardiography has a Consumer Health Vocabulary preferred term of heart ultrasound and Unified Medical Language System preferred name of echocardiography. Two thousand five hundred ninety four of the 3774 (69 %) annotations contained common CUIs between the Consumer Health Vocabulary and the ShARe test set. For these possible annotations, UTHealthCCB had the highest accuracy (75.0), followed by the majority sense baseline (73.2), and THCIB.B.1 (73.1) (Table 3).

**Table 3:** Accuracy of normalizing short forms with concept unique identifiers shared between the ShARe test set and the Consumer Health Vocabulary

Short form normalization system	Accuracy
UTHealthCCB.B.1	75.0
Majority Sense Baseline	73.2
THCIB.B.1	73.1
UTHealthCCB.B.2	70.4
LIMSI.1	69.6
TeamHealthLanguageLABS	50.9
WVU.1	50.1

## Discussion

We characterized the ShARe short form corpus, assessed participants’ systems, reported majority sense distributions for the most prevalent terms, assessed participants’ systems for each majority sense distribution category, evaluated the coverage of short form concepts against the Consumer Health Vocabulary, and assessed how well each participants’ system could normalize short forms with shared CUIs between the ShARe test set and the Consumer Health Vocabulary.

### Test corpus

We estimated that around 81 % of the short form annotations represent terms with none or low ambiguity (either one unique sense or two senses with a majority sense over 80 %); in contrast, about 19 % of the short form annotations are moderately to highly ambiguous (two senses with a majority sense between 50 and 80 %, or two senses with a majority sense less than 50 %). For example, the term ‘trach’ had two senses with a majority sense less than 80 %, requiring word sense disambiguation. For instance, in “now s/p trach”, ‘trach’ represents a *Therapeutic or Preventative Procedure* - C0040590: Tracheostomy Procedure. In “Assess for trach placement”, ‘trach’ represents a *Medical Device* - C0184159: Tracheostomy Tube. In the case of these polysemous (different, but related) senses, predicting C0040590: Tracheostomy Procedure instead of C0184159: Tracheostomy Tube may not necessarily result in a misunderstanding of the text by a patient due to level of shared concept similarity. In the case of the following homonymous (different and unrelated) sense example, ‘PT’ can represent C0949766: Physical therapy or C0030705: Patients. In such a case, it would be more important for a system to accurately select the correct sense for patient understanding of clinical text due to the lack of concept similarity.

### ShARe/CLEF eHealth challenge shared task 2 – system performances

We evaluated participant’s system performance for normalizing acronyms/abbreviations to Unified Medical Language System CUIs on the test set (Table 1). Compared to the majority sense baseline results, only the highest performing system by UTHealthCCB.1 showed improvement. Our majority sense baseline approach results (~70 % accuracy) are comparative to previously reported clinical majority sense baseline results (71 % accuracy) [53]. On the training set, THCIB reports 20 % of the short forms from a sentence input could not be mapped to CUIs using clinical Text Analysis and Knowledge Extraction System. We believe this demonstrates that out-of-the-box text normalization systems will perform moderately for normalizing short forms. Many participants incorporated clinical Text Analysis and Knowledge Extraction System pre-processing, conditional random field, and custom dictionaries from training data and online resources to develop their systems.

Systems with post-processing, sense disambiguation, and machine learning trained with natural language processing features can outperform a baseline short form normalization system. The system by UTHealthCCB used a hybrid approach incorporating rule-based and machine learning techniques and achieved an accuracy of 72 % which suggests short form normalization continues to be a challenging natural language processing research problem. Some teams developed an end-to-end system including short form boundary detection and normalization. This reason accounts for some variation in the number of predictions by participating systems.

### Sense prediction evaluation

We observed that of most short form terms with no or low ambiguity, over 80 % could be normalized with reasonable accuracies by participants’ systems. In contrast, short form terms with moderate or high ambiguity could be normalized with low to modest accuracy by participants’ systems (Fig. 1). This trend was consistent for all participating systems and approaches. This finding is not surprising, as we would expect some reduction in performance due to ambiguity.

### Consumer health vocabulary coverage

Of the 3774 ShARe/CLEF short form annotations, we observed most (94 %) short form annotations map to a CUI in the Unified Medical Language System i.e., only about 6 % of short form annotations were ‘CUI-less’, demonstrating excellent short form coverage. Over half (66 %) of the unique Unified Medical Language System CUIs in the test corpus also occurred in the Consumer Health Vocabulary implying that a substantial portion of short form concepts (34 %) could be considered for addition to the Consumer Health Vocabulary. About 52 % of the shared CUIs had a Consumer Health Vocabulary patient-friendly preferred name that differed from the Unified Medical Language System. In these cases, a patient-friendly alternative may be offered to a patient to improve understanding of clinical text. In contrast, some shared CUIs (48 %) have a Consumer Health Vocabulary preferred term that matched the Unified Medical Language System preferred term. In these cases a patient-friendly alternative may not be necessary. In future work, we plan to identify patient-friendly preferred terms for short form CUIs from the test corpus that did not occur in the Consumer Health Vocabulary and propose them for inclusion.

In terms of normalizing short forms with common CUIs between the Consumer Health Vocabulary and the ShARe test set, participant systems’ demonstrated moderate to reasonable accuracy suggesting promising results for supporting patient understanding of clinical text by replacing these concepts with a more lay term or linking these terms to web resources.

## Conclusion

We completed the 2013 ShARe/CLEF eHealth Challenge with the focus on creating resources that could be leveraged to develop technologies to aid patients’ understanding of his or her electronic medical record. For Task 2, we developed a reference standard for short form normalization with high inter-annotator agreement, adding an additional meta-data layer to the openly available ShARe corpus [48]. The natural language processing community demonstrated that a short form normalizer could be created with reasonably high accuracy; however, more work needs to be done to resolve short forms with moderate to high ambiguity. We demonstrated that more concepts could be added to the Consumer Health Vocabulary to support patient understanding of short forms used in clinical reports.

## Abbreviations

ADAM, Another Database of Abbreviations in Medline; ARGH, Acronym Resolving General Heuristic; CLEF, Conference & Labs of the Evaluation Forum; cTAKES, clinical Text Analysis and Knowledge Extraction System; CUI, Concept Unique Identifier; eHOST, extensible Human Oracle Suite of Tools; GENIA, Genome Information Acquisition; i2b2, Informatics for Integrating Biology and the Bedside; MedLEE, Medical Language Extraction and Encoding System; MIMIC, Multiparameter Intelligent Monitoring in Intensive Care II database; SNOMED CT, Systematized Nomenclature Of MEDicine Clinical Terms; MUC, Message Understanding Conference; SaRAD, Simple and Robust Abbreviation Dictionary; ShARe, Shared Annotated Resources; TREC, Text REtrieval Conference
